# Thorax CT findings in patients with Crimean-Congo hemorrhagic fever (CCHF)

**DOI:** 10.1186/s40064-016-3522-5

**Published:** 2016-10-21

**Authors:** Turan Aktaş, Fatma Aktaş, Zafer Özmen, Ayşegül Altunkaş, Turan Kaya, Osman Demir

**Affiliations:** 1 Department of Pulmonary Medicine, Gaziosmanpaşa University, School of Medicine, Tokat, Turkey; 2 Radiology Department, Gaziosmanpaşa University, School of Medicine, Tokat, Turkey; 3 Infection Disease and Clinical Microbiology, Tokat State Hospital, Tokat, Turkey; 4 Statistics Department, Gaziosmanpaşa University, School of Medicine, Tokat, Turkey

**Keywords:** Crimean-Congo hemorrhagic fever, Thorax CT, Pleural effusion, Alveolar infiltration, Parenchymal infiltration

## Abstract

**Purpose:**

Crimean-Congo hemorrhagic fever (CCHF) is a zoonotic viral disease with high mortality. The agent causing CCHF is a Nairovirus. The virus is typically transmitted to humans through tick bites. CCHF is a life-threatening disease observed endemically over a wide geographical regions in the world and a little known about pulmonary findings in CCHF patients.

**Methods:**

The patients that were admitted and diagnosed with CCHF between April 2010 and September 2015 were examined. Patients’ medical records were then evaluated retrospectively. Patients who underwent thorax CT evaluation based on the clinical findings at the time of admission and/or during the hospital stay were included in the study. Patients’ laboratory test results and thorax CT findings for respiratory assessment along with demographic characteristics.

**Results:**

Forty patients diagnosed with CCHF that underwent thorax CT based on their indications were included in the study. Twenty-seven patients (62.5 %) were male with a mean age of 55.22 ± 19.84 years. According to these results, the three most common thorax CT findings were parenchymal infiltration [32 patients (80 %)], pleural effusion [31 patients (77.5 %)], and alveolar infiltration [28 patients (70 %)]. Moreover, we determined that the most frequently seen radiological findings often occurred bilaterally.

**Conclusions:**

There is still not enough information regarding this life-threatening disease. We also would like to emphasize that both direct radiography and thorax CT are highly successful in detecting frequently encountered radiological findings such as pleural effusion, alveolar hemorrhage, and parenchymal infiltration that indicate pulmonary involvement.

## Background

Crimean-Congo hemorrhagic fever (CCHF) is a zoonotic viral disease with a high mortality. CCHF is caused by Nairovirus, which is an RNA-type virus from the Bunyaviridae family. It is generally transmitted through the bite of vectors, such as ticks. Although rare, infection with CCHF has also been reported in health care professionals as a result of contact with blood and blood products. Non-specific symptoms such as fever, malaise, loss of appetite, nausea, vomiting, and myalgia might occur after contact with infected blood. In addition to these non-specific symptoms, bruising, petechiae, vaginal bleeding, and epistaxis as well as signs of intracranial, gastrointestinal, and alveolar hemorrhage that may be life threatening and often responsible for mortality might occur. The mortality rate due to CCHF ranges from 3 to 80 %. The disease was named Crimean-Congo hemorrhagic fever after the first case of the disease was seen in Crimea in 1944 and a similar case was reported in Congo 12 years later. Furthermore, it is endemic in many parts of the world. In literature, the CCHF cases from about 30 countries encompassing Africa, Asia, Eastern Europe, and the Middle East have been reported, with the number of cases increasing each year (Karti 2009; Ergönül 2006).

Despite the increasing number of cases in Turkey and other endemic areas in the world over the years, the number of studies related to CCHF’s lung findings is very limited. In particular, very little is known about the effects of extensive, systemic inflammation and vascular damage symptoms on pathophysiological changes in the lung tissue as well as its clinical outcome. Moreover, not much is known about CCHF’s lung findings in specific and advanced imaging techniques, such as computed tomography (CT), and their clinical importance and benefits in follow-up. In this study, we evaluated patients that were diagnosed with CCHF and whose thorax CT scans were taken due to pulmonary symptoms. We aimed to demonstrate the relationship between clinical follow-up findings and laboratory values among these patients.

## Methods

The patients that were admitted to Tokat State Hospital Emergency Department and Infectious Diseases and Clinical Microbiology Department and diagnosed with CCHF between April 2010 and September 2015 were examined. Also Tokat State Hospital Infection Disease and Clinical Microbiology Department granted us access to all patients’ datas and then patients’ medical records were evaluated retrospectively. Patients who underwent thorax CT evaluation based on the clinical findings at the time of admission and/or during the hospital stay were included in the study. Ethical approval was obtained from ethics committee of Tokat Gaziosmanpaşa University. Patients’ laboratory test results, chest X-ray, and thorax CT findings for respiratory assessment along with demographic characteristics such as age, gender, occupation, place of residence, tick exposure, smoking history, clinical diseases, and medical history laboratory were evaluated.

Blood samples were taken from the patients at the time of admission for diagnostic purposes and were sent to Ankara Refik Saydam Hıfzısıhha Central Virology Laboratory. The diagnosis of CCHF was then made based on the enzyme-linked immunosorbent assay (ELISA) or by detection of viral genome by polymerase chain reaction (PCR).

### Statistical analysis

Data was expressed as mean ± SD or frequency and percent. Independent sample *t* test or one-way analysis of variance were used in order to compare the continuous normal data between groups. The Turkey HSD test was used for post hoc comparisons between the pair-wise groups. Furthermore, the Kaplan–Meier method was used for survival probability. A *p* value <0.05 was considered to be significant and analyses were performed using SPSS 19 (IBM SPSS Statistics 19, SPSS Inc., an IBM Co., Somers, NY).

## Results

A total of 563 CCHF patients that were admitted to Tokat State Hospital Emergency Department and Infectious Diseases and Clinical Microbiology Department between April 2010 and September 2015 were evaluated for this study. Forty patients that underwent thorax CT based on their indications were included in the study. Thirteen patients (37.5 %) were female with a mean age of 51.15 ± 15.91 years, while 27 patients (62.5 %) were male with a mean age of 55.22 ± 19.84 years. There was no significant difference between males and females in terms of mean age. Patients’ demographic characteristics are given in detail in Table 1.

**Table 1:** Demographic and clinical features of CCHF patients in the study

Variables	Values	n (% or ±SD)
Age	<65 years	26 (65 %)
	≥65 years	14 (35 %)
Gender	Female (mean age 51.15 year ± 15.91)	13 (32.5 %)
	Male (mean age 51.15 year ± 15.91)	27 (67.5 %)
Smoking habit	Non-smoker	15 (37.5 %)
	Smoking	24 (60 %)
	Ex-smoker	1 (2.5 %)
Symptoms	Fever	39 (97.5 %)
	Fatigue	36 (90 %)
	Myalgia	17 (42.5 %)
	Vomiting	9 (22.5 %)
	Enteritis	9 (22.5 %)
	Abdominal pain	13 (32.5 %)
	Inappetence	4 (10 %)
	Eruptions	8 (20 %)
	Bleeding	11 (27.5 %)
	Cough	9 (22.5 %)
	Somnolence	13 (32.5 %)
	Headache	10 (25 %)
	Dizziness	9 (22.5 %)
Hospitalization place	Inpatient service unit	21 (52.5 %)
	Critical care unit	5 (12.5 %)
	Both	14 (35 %)
Mean hospitalization time (day)	In inpatient service unit (day)	10.17 (±8.18)
	In critical care unit (day)	8.73 (±6.75)
Treatment	Supportive treatment	35 (87.5 %)
	Supportive and antiviral treatment	5 (12.5 %)
Survival	Alive	26 (65 %)
	Death	14 (35 %)

The comparative evaluation of thorax CT findings between the gender groups showed that the only significant increase was in pulmonary artery diameter (*p* < 0.05). In addition, among the reported symptoms, only the presence of petechiae and ecchymotic rash were significantly different among gender groups (*p* < 0.05). There were no other significant relationships between gender and patients’ clinical, radiological, and laboratory parameters.

Patients were divided into two groups based on their age, being composed of under and over the age of 65. Twenty-six patients (65 %) were over the age of 65 years. According to our results, symptoms of myalgia were more common in patients in the <65 age group and the difference between the age groups was statistically significant. In addition, 10 of the 11 patients (38.5 %) with minor bleeding (such as epistaxis, menorrhagia, and mucosal bleeding) were in the >65 age group. These findings were found to be statistically significant and no other significant associations were detected between age and other findings.

Laboratory evaluation included biochemical, hematologic, coagulation, and inflammatory parameters. Furthermore, mean and standard deviation values of all parameters were calculated. The laboratory values of the patients are shown in detail in Table 2.

**Table 2:** The laboratoury findings of CCHF patients in the study

Parameters	Mean	SD±	Normal range
Alkaline phosphatase (ALP)	178.72	156.07	35–270 U/L
Albumin	3.51	0.39	3.5–5 mg/dL
Alanine aminotransferase (ALT)	413.64	767.18	7–35 U/L
Aspartate aminotransferase (AST)	1330.07	2811.95	10–38 U/L
Blood uremia nitrogen (BUN)	35.53	33.27	8–23 mg/dL
Direct bilirubin	1.14	2.10	0.01–0.3 mg/dL
Gamma glutamyl transferase (GGT)	254.07	266.50	5–61 U/L
Creatine kinase (CK)	1303.55	1747.15	0–170 U/L
C-reactive protein (CRP)	76.95	82.60	0–5 mg/L
Calcium	8.13	0.78	8.8–10.6 mg/dL
Creatinine	1.69	1.58	0.6–1.09 mg/dL
Lactate dehydrogenase (LDH)	1798.62	2153.65	135–214 U/L
Total bilirubin	1.64	2.49	0.01–1.2 mg/dL
Uric acid	6.11	3.78	3.5–7.2 mg/dL
White blood cell	3.56	3.77	4.5–10 10^3^/µL
Neutrophile	2.64	3.40	2–8.9 10^3^/µL
Lymphocyte	0.67	0.48	1. 3–3.35 10^3^/µL
Monocyte	0.25	0.27	0.25–0.85 10^3^/µL
Red blood cell	4.67	0.86	4.5–5.9 10^6^/mL
Red cell distribution width (RDW)	14.33	3.32	12.1–14.3 %
Hemoglobin	13.00	2.70	13.5–17.5 gr/dl
Haematocrit	38.01	7.44	41–53 %
Platelet	33.26	27.73	150–400 10^3^/μL
Platelet distribution width (PDW)	22.80	5.63	10.1–16.1 %
Mean platelet volume (MPV)	11.36	2.39	9.1–11.9 fL
Partial thromboplastin time (%PTT)	79.55	29.00	70–120 %
Partial thromboplastin time (PTT)	15.86	5.66	10.1–14.9 s
International normalization ratio (INR)	1.44	0.55	0.8–1.2
Activated partial thromboplastin time (APTT)	67.43	28.09	23–35 s
D-dimer	6.92	4.59	0–0.5 mg/dL
Fibrinogen	284.03	111.10	202–430 mg/dL
Ferritin	16,860.40	19,272.16	20–400 µg/L
Procalcitonin	2.75	4.65	0–0.05 µg/L
Sedimentation	25.35	26.70	0–20 mm/h

*SD* standard deviation

**Fig. 1:**
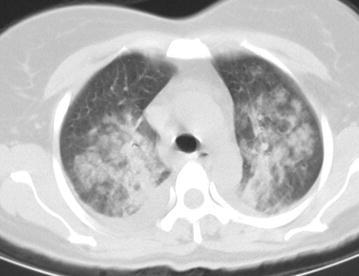
Fourty years old female patient with the alveolar densities and ground glass opacities (alveolar hemorrhage) were seen in computerized tomography (CT) scanning of the chest

**Fig. 2:**
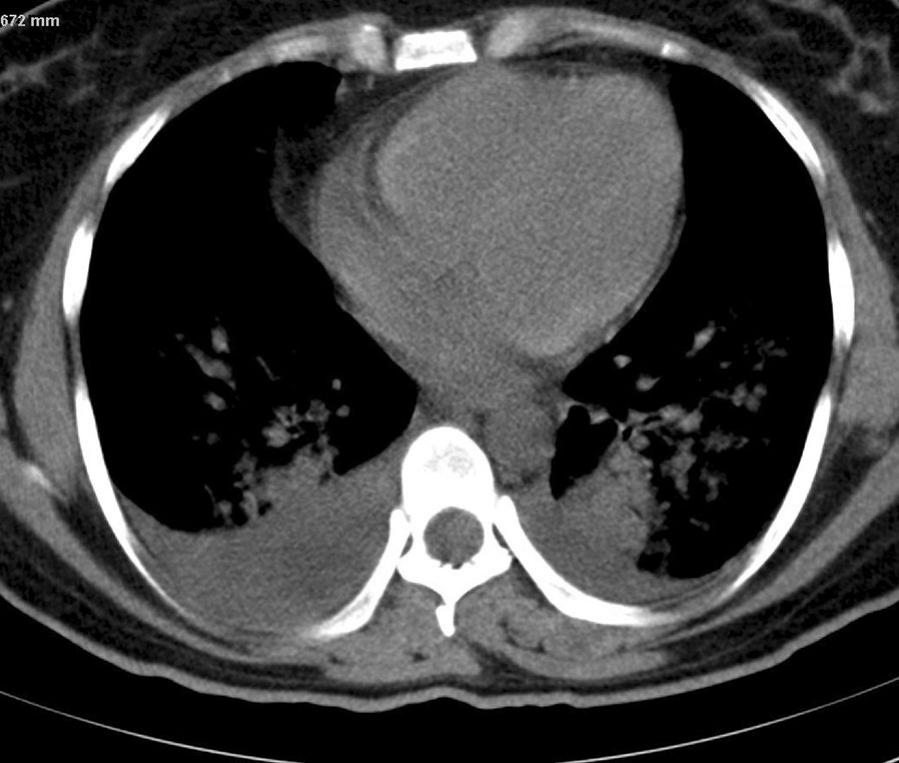
The same patient in Fig. 1 with bilateral pleural effusion is seen in CT scanning of the chest

**Fig. 3:**
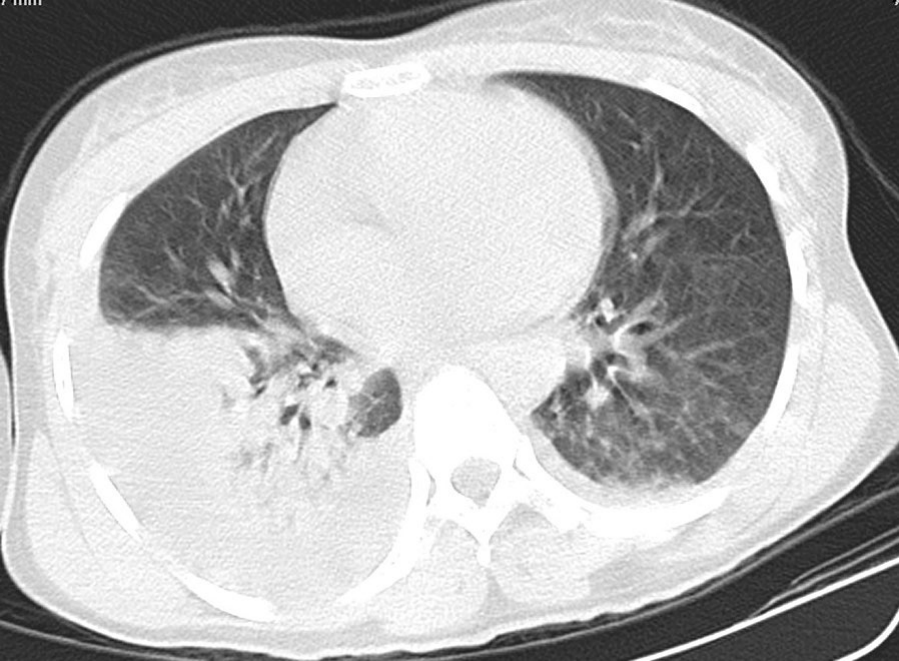
Twenty-six years old female patient with bilateral pleural effusion and consolidation with air bronchograms at the right lower lobe are seen in CT scanning of the chest

The patients’ radiological chest CT findings were evaluated. The radiological assessment showed a total of 13 thorax CT findings, with one patient having multiple findings. According to these results, the three most common thorax CT findings were parenchymal infiltration [32 patients (80 %)], pleural effusion [31 patients (77.5 %)], and alveolar infiltration [28 patients (70 %)]. Meanwhile, the least common CT findings were bronchiectasis [3 patients (7.5 %)] and mediastinal lymphadenopathy [2 patients (5 %)]. Moreover, we determined that the most frequently seen radiological findings such as parenchymal infiltration, alveolar infiltration, and pleural effusion often occurred bilaterally (Figs. 1, 2 and 3). The unilateral pathology was seen less frequently and most of these cases occurred in radiographic findings of the right lung. Patients’ thorax CT findings are summarized in detail in Tables 3 and 4.

**Table 3:** Thoracic CT findings of CCHF patients in the study

Thorax CT findings	There n (%)	None n (%)
Alveolar infiltration	28 (70 %)	12 (30 %)
Parenchymal infiltration	32 (80 %)	8 (20 %)
Pleural effusion	31 (77.5 %)	9 (22.5 %)
Atelectasis	26 (65 %)	14 (35 %)
Small-airway involvement	13 (32.5 %)	27 (67.5 %)
Enlargement of pulmonary artery	27 (67.5 %)	13 (32.5 %)
Cardiomegaly	6 (15 %)	34 (85 %)
Pericardial effusion	5 (12.5 %)	35 (87.5 %)
Ground-glass opacity	23 (57.5 %)	17 (42.5 %)
Consolidation	11 (27.5 %)	29 (72.5 %)
Mediastinal/hilar lymphadenopathies	2 (5 %)	38 (95 %)
Chronic fibrotic changes	9 (22.5 %)	31 (77.5 %)
Bronchiectasis	3 (7.5 %)	37 (92.5 %)
Pulmonary nodule	8 (20 %)	32 (80 %)

**Table 4:** Locations of the radiological findings in CCHF patients with assessment of thoracic CT

Thorax CT findings	Location	n (%)
Alveolar infiltration	Bilateral	19 (47.5 %)
	Right	7 (17.5 %)
	Left	2 (5 %)
	None	12 (30 %)
Parenchymal infiltration	Bilateral	20 (50 %)
	Right	9 (22.5 %)
	Left	3 (7.5 %)
	None	8 (20 %)
Pleural effusion	Bilateral	15 (37.5 %)
	Right	14 (35 %)
	Left	2 (5 %)
	None	9 (22.5 %)
Atelectasis	Bilateral	22 (55 %)
	Right	4 (10 %)
	None	14 (35 %)
Small-airway involvement	Bilateral	9 (22.5 %)
	Right	2 (5 %)
	Left	2 (5 %)
	None	27 (67.5 %)
Enlargement of pulmonary artery	Bilateral	27 (67.5 %)
	None	13 (32.5 %)
Ground-glass opacity	Bilateral	17 (42.5 %)
	Right	6 (15 %)
	None	17 (42.5 %)
Consolidation	Bilateral	8 (20 %)
	Right	1 (2.5 %)
	Left	2 (5 %)
	None	29 (72.5 %)
Mediastinal/hilar lymphadenopathies	Bilateral	2 (5 %)
	None	38 (95 %)

In this study we also evaluated patients’ survival, factors affecting survival, and the association between survival and patients’ other clinical, laboratory, and radiological parameters. A total of 26 patients (65 %) survived, while 14 patients (35 %) did not survive. We also determined that presence of symptoms such as rash, sleepiness, and dizziness at the time of admission was significantly associated with survival (*p* < 0.05). Parenchymal and alveolar infiltration, pleural effusion, and atelectasis that were significantly associated with survival and were more frequently seen in the non-survival group were observed bilaterally in thorax CT scans. Both the patients’ survival and the relationship between thorax CT findings are presented in detail in Table 5. Moreover, the patients’ mortality rate was also calculated and found to be 35 %.

**Table 5:** The comparison between survival and thoracic CT findings of CCHF patients

Thorax CT findings	Survival		*p*
	n (%) Alive	n (%) Death	
Atelectasis	14 (53.8)	12 (85.7)	*0.044*
Parenchymal infiltration	18 (69.2)	14 (100)	*0.020*
Alveolar infiltration	15 (57.7)	13 (92.9)	*0.021*
Cardiomegaly	3 (11.5)	3 (21.4)	0.403
Enlargement of pulmonary artery	17 (65.4)	10 (71.4)	0.697
Small-airway involvement	9 (34.6)	4 (28.6)	0.697
Pericardial effusion	2 (7.7)	3 (21.4)	0.210
Mediastinal/hilar lymphadenopathies	1 (3.8)	1 (7.1)	0.648
Pulmonary nodule	6 (23.1)	2 (14.3)	0.507
Ground-glass opacity	13 (50)	10 (71.4)	0.191
Consolidation	6 (23.1)	5 (35.7)	0.393
Chronic fibrotic changes	9 (34.6)	0 (0)	*0.012*
Bronchiectasis	2 (7.7)	1 (7.1)	0.950
Pleural effusion	17 (65.4)	14 (100)	*0.012*

Statistically significant *p* values are in Italic

Patients that were diagnosed with CCHF based on their radiological findings underwent a chest X-ray (CXR). The presence of pathological findings on CXR and pleural effusion, parenchymal and alveolar infiltration, and bronchiectasis seen on thorax CT scans were significantly associated. Other findings were only detected on thorax CT scans and not on CXR. The relationship between CXR and thorax CT findings are given in detail in Table 6.

**Table 6:** The comparison between the radiological findings of chest X-ray and thoracic CT in CCHF patients

Thorax CT findings	CXR		*p*
	CXR findings (−) n (%)	CXR findings (+) n (%)	
Atelectasis	3 (50)	23 (67.6)	0.403
Parenchymal infiltration	2 (33.3)	30 (88.2)	*0.002*
Alveolar infiltration	1 (16.7)	27 (79.4)	*0.002*
Cardiomegaly	1 (16.7)	5 (14,7)	0.901
Enlargement of pulmonary artery	3 (50)	24 (70.6)	0.321
Small-airway involvement	0 (0)	13 (38.2)	0.065
Pericardial effusion	0 (0)	5 (14,7)	0.315
Mediastinal/hilar lymphadenopathies	0 (0)	2 (5.9)	0.542
Pulmonary nodule	1 (16.7)	7 (20.6)	0.825
Ground-glass opacity	3 (50)	20 (58.8)	0.687
Consolidation	0 (0)	11 (32.4)	0.102
Chronic fibrotic changes	1 (16.7)	8 (23.5)	0.711
Bronchiectasis	1 (16.7)	2 (5.9)	0.355
Pleural effusion	1 (16.7)	30 (88.2)	*<0.001*

Statistically significant *p* values are in Italic

## Discussion

CCHF is a viral zoonotic disease. It is endemic in many geographic regions of the world and is seen at the end of spring as well as during the summer months and can cause life-threatening bleeding. There is still not much information regarding the etiopathogenesis, clinical follow-up, and treatment; however it is known that CCHF is transmitted through tick bites and that Nairovirus is the main factor that causes intense systemic inflammatory response in the entire body. As a result of this inflammatory response, non-specific signs such as fever, fatigue, and myalgia occur in the acute phase. During the advanced stages of the disease, low platelets can cause serious spontaneous bleeding and even neurological symptoms.

In our study, the male to female ratio was about 2–1 (27/13), which was similar to other studies in the literature. In our country and region, mostly men work in occupations such as farming, animal husbandry, veterinary medicine, and the military which require a frequent outdoor presence. Therefore, men are more likely to come into contact with ticks that are vectors for CCHF transmission.

CCHF is a blood borne and contagious disease. Therefore, there are many studies in literature focusing on blood, other body fluids, and evaluating laboratory findings. Some studies have reported that there is a strong relationship between survival and parameters such as d-dimer, fibrinogen, platelets, leukocytes, neutrophil count, and muscle and liver enzymes (Karti 2009; Ergönül 2006; Watts et al. 1988; Schwarz et al. 1997). Similar to studies in literature, survival was significantly associated with parameters such as leukocytes, neutrophils, and platelet counts in the blood count test; ALT, AST, BUN, direct bilirubin, CK, creatinine, LDH, total bilirubin, uric acid in the biochemical tests; fibrinogen, d-dimer, ferritin, PTT, and APTT in the coagulation test (*p* < 0.05).

The number of studies focusing on lung findings in CCHF is very limited. In all of these studies, lung evaluations were performed based on the plain radiography and to the best of our knowledge, there are no other studies performed with thorax CT. In 2007 a study from Russia by Sannikova et al. was the first study that evaluated lung findings in CCHF. In their study, lung findings of 283 patients with acute respiratory distress syndrome (ARDS) were defined and it was noted that these symptoms could be seen in all periods, most notably in the hemorrhagic phase. In addition, they concluded that ARDS symptoms that may occur throughout the course of the disease are closely associated with systemic inflammation and that this clinical condition may accompany hemorrhagic symptoms (Sannikova et al. 2007). In 2012, a Turkish study by Bilgin et al. evaluated lung findings of 128 CCHF patients, retrospectively. The most frequently detected pulmonary symptoms were cough, sputum, and chest pain. In addition, they reported that there was no significant difference between patients with and without radiological findings in terms of survival. In the majority of patients (65.5 %) that had radiological findings, these findings were bilateral. However, the study only evaluated direct radiography findings and abnormal findings that were detected included infiltration, hilar pathology, interstitial pathology, pleural thickening, and pleural effusion (Bilgin et al. 2014). Another study that evaluated lung findings of CCHF was done by Doğan et al. in 2011. The study assessed demographic, clinical, and radiological parameters as well as pulmonary symptoms of 108 patients diagnosed with CCHF. Similar to the aforementioned studies, the radiological assessments were done based on the direct radiography findings. Abnormal findings in direct radiography were not detected in 66.7 % of patients. On the other hand, the majority of the radiological findings detected in direct radiography were unilateral (Dogan et al. 2011). In literature, there are single case reports for both pediatric and adult cases that used direct radiography and thorax CT to evaluate lung findings. Pleural effusion was found in almost all case reports and is the most frequent as well as the most well defined radiological finding in CCHF patients in literature. Although, the precise pathophysiological mechanisms of pleural effusion are not known, it is widely thought to develop as a result of widespread systemic inflammation. However, it is recommended that differential diagnosis of hemothorax should be included. It is suggested that the pleural effusion that develops in the course of the disease is not associated with hemothorax (Schnittler and Feldmann 2003; Feldman et al. 2003; Tanir et al. 2009). In Doğancı et al.’s case report from Turkey, a young adult diagnosed with CCHF had an alveolar hemorrhage at the time of admission to the hospital, which regressed dramatically on day 5 after ribavirin and supportive treatment (Doganci et al. 2008). Although the authors suggested that excessive inflammatory response was the reason, it remains unclear whether thrombocytopenia and/or vascular injury might have been the main factors in the pathophysiology.

There are 4 stages of clinical follow-up in CCHF: incubation phase, pre-hemorrhagic phase, hemorrhagic phase, and convalescent phase (Ergönül 2008; Bakir et al. 2005). In our study, the patients underwent thorax CT for further diagnosis due to the emergence of new findings in the pre-hemorrhagic and hemorrhagic phase at the time of admission and/or during the clinical follow-up. Radiological findings suspected to be pulmonary pathologies of CCHF were seen in all patients. Inadequacies in the differential diagnosis and further investigations may occur, especially in the pre-hemorrhagic phase because patients come in with symptoms similar to the common cold. In particular, the most frequently seen thorax CT findings, such as parenchymal infiltration, alveolar infiltration, pleural effusion, and atelectasis, are significantly related with survival which in turn, emphasizes the importance of these parameters in clinical follow-up. In addition, if these findings develop in advanced stages of the disease, such as hemorrhagic and convalescent phases, they might serve as indicators of poor prognosis.

The presence of fibrotic changes in thorax CT findings was also found to be significantly related to survival. Interestingly, all nine patients that developed fibrosis survived. This can be explained by insufficient inflammatory response in patients with fibrotic changes. However, this view is based exclusively on our clinical data and further studies at the cellular and molecular level are required.

In our study, we also compared direct radiography and thorax CT in terms of their success in revealing radiologic findings. In all previous studies only direct radiography was used for the assessment of the lung and none of them felt the need to use thorax CT for advanced diagnosis. Therefore, we wanted to evaluate the adequacy of direct radiography in the assessment of the lung findings in patients with CCHF. The direct radiography can detect the radiological findings most frequently seen in thorax CT such as pleural effusion, alveolar, and parenchymal infiltration. The correlation between direct radiography and thorax CT in terms of detection of radiologically similar findings was statistically significant (*p* < 0.05). This suggests that it is more appropriate to use simple, cheap, and easily accessible direct lung radiography in the initial diagnosis of CCHF, however in cases with additional pathological findings, differential diagnosis or the absence of clinical improvement thorax CT should be used for further examination in clinical follow-up. Therefore, CXR should be considered especially in patients in the hemorrhagic phase for detection of pulmonary pathologies such as alveolar hemorrhage and pleural effusion, while thorax CT should be considered for further evaluation. Moreover, it should be deliberated that the pre-hemorrhagic phase, in which the symptoms emerge, will be followed by the hemorrhagic phase when the clinical manifestations could occur, and thus radiological assessment of the lungs should be carried out after the admission to the hospital. This radiological evaluation will be very useful in terms of calculating both the survival and the clinical course of the disease.

In conclusion, there is still not enough information regarding this life-threatening disease that is endemic in many geographic regions of the world. Furthermore, the number of deaths due to CCHF is increasing every year. In CCHF, the intense inflammatory response affects all systems, including the circulation and hematological system and clinical implications of the liver as well. However, information regarding effects on the respiratory system is very limited. In particular, the information regarding the radiologic evaluations and findings for this disease is insufficient. In cases with life-threatening clinical conditions it is important to evaluate radiological findings in the respiratory system by using direct radiography and by using thorax CT when differential and advance diagnosis is needed. We also would like to emphasize that both direct radiography and thorax CT are highly successful in detecting frequently encountered radiological findings such as pleural effusion, alveolar hemorrhage, and parenchymal infiltration that indicate pulmonary involvement and have a negative effect on survival and therefore should be used in the evaluation of patients with CCHF.
